# RAGE-specific single chain Fv for PET imaging of pancreatic cancer

**DOI:** 10.1371/journal.pone.0192821

**Published:** 2018-03-12

**Authors:** Hye-Yeong Kim, Xiaolei Wang, Rui Kang, Daolin Tang, Brian A. Boone, Herbert J. Zeh, Michael T. Lotze, W. Barry Edwards

**Affiliations:** 1 Molecular Imaging Laboratory, Department of Radiology, University of Pittsburgh, Pittsburgh, Pennsylvania, United States of America; 2 Department of Surgery, University of Pittsburgh Cancer Institute, Pittsburgh, Pennsylvania, United States of America; 3 Department of Bioengineering, University of Pittsburgh Cancer Institute, Pittsburgh, Pennsylvania, United States of America; 4 Department of Immunology, Hillman Cancer Center, University of Pittsburgh Cancer Institute, Pittsburgh, Pennsylvania, United States of America; University of Miami School of Medicine, UNITED STATES

## Abstract

Noninvasive detection of both early pancreatic neoplasia and metastases could enhance strategies to improve patient survival in this disease that is notorious for an extremely poor prognosis. There are almost no identifiable targets for non-invasive diagnosis by positron emission tomography (PET) for patients with pancreatic ductal adenocarcinoma (PDAC). Over-expression of the receptor for advanced glycation end products (RAGE) is found on the cell surface of both pre-neoplastic lesions and invasive PDAC. Here, a RAGE-specific single chain (scFv) was developed, specific for PET imaging in syngeneic mouse models of PDAC. An anti-RAGE scFv conjugated with a sulfo-Cy5 fluorescence molecule showed high affinity and selectivity for RAGE expressing pancreatic tumor cells and genetically engineered KRAS^G12D^ mouse models of PDAC. An *in vivo* biodistribution study was performed with the ^64^Cu-radiolabled scFv in a syngeneic murine pancreatic cancer model, demonstrating both the feasibility and potential of an anti-RAGE scFv for detection of PDAC. These studies hold great promise for translation into the clinic.

## Introduction

Pancreatic ductal adenocarcinoma (PDAC) is the fourth leading cause of cancer mortality in the U.S. and associated with an extremely poor clinical outcome. The 5-year patient survival rate for all patients in aggregate is less than 5%, thought to be due to late diagnosis, early metastasis, and resistance to chemotherapy [1]. Unlike other solid tumors, treatment options for patients with PDAC are limited. Surgical removal of the tumor at an early stage before invasion is the only currently available therapy with curative intent [2]. Early detection of PDAC, or its precursor lesion, pancreatic intraepithelial neoplasia (PanIN), could potentially improve treatment outcomes [2]. Non-invasive diagnostic imaging by positron emission tomography (PET) is an ideal tool for assessing the functional tumor status within the pancreas. Fluoro-deoxyglucose (FDG) imaging has been widely used for diagnosis of various cancers and diseases, revealing Warburg biology within tumors displaying prominent glycolytic metabolism [3]. FDG is limited in its application for early diagnosis and differentiation of pre-invasive PDAC from frankly invasive pancreatic cancer [4]. Detection of the receptor for advanced glycation end products (RAGE), found early in the glycolytic and autophagic switch during carcinogenesis, could provide important information about the disease stage and reveal tumor progression. This would allow image-guided and targeted therapy in patients with pancreatic cancer. We investigated the sensitivity of a novel anti-RAGE scFv antibody applied for molecular imaging of pancreatic cancer.

RAGE is overexpressed in human pancreatic tumors but not in adjacent normal ducts. Recent studies have shown that RAGE is a critical promoter in transition of premalignant epithelial precursors (PanIN) to invasive cancer (PDAC) [5–7]. RAGE is a member of the immunoglobulin gene superfamily, expressed within the Major Histocompatability (MHC) Class III region that binds multiple ligands, including advanced glycation end products (AGEs), S100/calgranulins, amphoterin/HMGB1 (high-mobility group box-1 chromosomal protein), Mac 1, DNA, and amyloid β-peptides [8–11]. Up-regulation of RAGE expression following ligand binding are associated with tumors in the brain, breast, colon, prostate, skin, liver, ovarian, and pancreas [12]. The cumulative evidence from both genetically engineered mouse models and human tumor histologic studies demonstrates that high expression of RAGE is directly linked to pancreatic tumorigenesis and chemoresistance, indicating that RAGE is both a novel biomarker as well as a target for pancreatic cancer.

Here, we developed a RAGE specific antibody fragment (single chain Fv) for detection of RAGE expressing pancreatic tumor. The small size of scFv (~25 kDa) is superior to the greater size of intact antibody (~150 kDa) allowing rapid systemic clearance and enabling deep tumor tissue penetration, which is beneficial for same day diagnostic studies. A fluorescent dye (Cy5) labeled anti-RAGE scFv was first synthesized, showing high affinity binding to murine RAGE (mRAGE) and no evidence of internalization in viable pancreatic carcinoma cell lines. Moreover, anti-RAGE scFv antibodies successfully visualized RAGE expression in genetically engineered KRAS^G12D^ mouse pancreatic tissues. *In vivo* biodistribution studies using a ^64^Cu-labeled scFv antibody fragment in an syngeneic mouse model demonstrated receptor specific uptake in RAGE-enriched tissues. This is the first report of pancreatic cancer-associated *in vivo* RAGE detection using an anti-RAGE scFv, suggesting feasibility for molecular imaging of patients with pancreatic cancer.

## Materials and methods

### Production of RAGE-specific scFv antibodies

The pIT2 vector was used as a parent vector for a phagemid construction. The nucleotide sequence of anti-RAGE scFv (3B4) was derived from the clone-3B4 [13]. To generate a non-binding control, M4, an scFv was designed by grafting the complementarity determining regions of clone-3B4 onto the 4D5 framework, which has the V_H_ and V_L_ reversed relative to 3B4. Nucleotide sequences of RAGE scFvs were spanned by restriction enzyme sites NcoI and NotI and synthesized for cloning into pUC57 vector from GenScript. The synthesized sequences were digested and purified using agarose gel electrophoresis, and ligated into NcoI and NotI digested phagemid pIT2 using T4 DNA ligase. Following transformation, positive clones were selected by colony PCR screening and further confirmed by DNA sequencing (Genewiz) using purified phagemids. In these scFvs, both a His6-tag and a myc-tag were inserted at the *C*-terminus for affinity protein purification and further analysis. The plasmid was transformed into BL21 *E*. *coli* for protein expression. The production of the scFvs was induced by addition of IPTG (400 μM, 30°C, 18 h) and isolated using Ni-NTA Agarose (Qiagen, Cat # 30210). The isolated scFvs were quantified by BCA protein assay and characterized by SDS-PAGE with Coomassie blue staining.

### Conjugations of sulfo-Cy5-NHS and p-SCN-Bn-NOTA with scFvs

scFvs (3B4 and M4) were reacted with 25-fold molar excess sulfo-Cy5-NHS (Lumiprobe) in 0.1 M NaHCO_3_ (pH ~9) at 4°C overnight. The scFv conjugates were purified by size exclusion chromatography (SEC) (P-10, GE Healthcare) using PBS as an eluent. The pure fractions of scFv-Cy5 were confirmed using SEC column chromatography by monitoring peaks at OD214 nm and OD646 nm. The concentration of sulfo-Cy5 of scFv conjugates was calculated by measuring absorption at 646 nm (ε = 271,000 M^-1^cm^-1^). The metal chelator, p-SCN-Bn-NOTA [S-2-(4-Isothiocyanatobenzyl)-1,4,7-triazacyclononane-1,4,7-triacetic acid] (Macrocyclics), was conjugated with scFvs in a similar reaction condition used for the optical dye conjugation reaction; 0.1 M ammonium acetate (pH 6.5) was used for SEC (P-10, GE Healthcare) buffer exchange. NOTA-conjugated scFv antibodies were analyzed by MALDI-TOF mass spectrometry (Voyager), and sinapinic acid was used as a matrix. Protein concentration was measured using BCA protein assay, and the protein purity and size were confirmed by SDS-PAGE and SEC.

### Measurement of affinity and kinetics using SPR

A Biacore X100 (GE Healthcare) was used for binding kinetic study of purified scFvs. To evaluate the binding affinity of scFvs, recombinant mouse RAGE Fc Chimera (R&D System, 1179-RG-050) was immobilized on a CM5 sensor chip (GE Healthcare) via an amine coupling (~2,000 RU). The carboxyl groups of the reference cell were activated and quenched with aminoethanol. The anti-RAGE-Mab, scFvs and conjugates were diluted in HBS-EP buffer (10 mM HEPES, 150 mM NaCl, 3 mM EDTA, and 0.005% surfactant P20, pH 7.4) and flowed over the surface for 180 s at a rate of 30 μL/min followed by dissociation for 600 seconds. After each sample injection, the surface was regenerated with consecutive injections of 5 μL of 0.3% SDS solution and 3.3 μL 50 mM NaOH. All sensorgrams were double referenced by subtracting the surface effect from the control flow cell and the buffer effect from the blank buffer. The calculated kinetic values *k*_a_, *k*_d_, and K_*D*_ were obtained using Biacore X100 Evaluation Software (GE Healthcare) assuming the Langmuir 1:1 binding model. The fitted curves are superimposed on the binding isotherms.

### Tissue immunofluorescence microscopy

The experimental protocols were approved by the Animal Care and Use Committee at the University of Pittsburgh. Mice were housed in a pathogen free environment in groups of five per cage with light/dark cycle of 12 hours. Female C57/BL6 wild-type mice (10–12 week) were purchased from Taconic Farms (Hudson, NY). For the syngeneic orthotopic pancreatic cancer model, mice underwent a limited laparotomy and were injected with Panc02 cells (1 x 10^6^) into the tail of the pancreas. Sham mice were subjected to the same operation and injected with PBS only. Animals were sacrificed following 4 weeks at which time abdominal tumors were palpable. We also utilized a genetically engineered model of KRAS driven pancreatic cancer (KC, Pdx1-Cre:Kras^G12D^) which were purchased from the National Cancer Institute Mouse Repository. KC mice and RAGE^-/-^mice were crossed to generate KCR mice (Pdx1-Cre:Kras^G12D:^ RAGE^-/-^). KC and KCR mice were sacrificed at 40–42 weeks [5]. Tissues were quickly frozen in cold hexane at -60°C and embedded in Tissue-Tek optimal cutting temperature (OCT) compound (Andwin Scientific). The frozen tissue was cut into 8 μM thickness sections on a cryostat (Microm HM 500OM Cryostat). Tissue sections on the glass slides were kept at– 80°C and hydrated in PBS followed by fixation in acetone at -20°C.

The sections were incubated with 3% horse serum blocking solution in PBS for 1 hr at room temperature and washed with PBST (0.1% Tween in PBS). For tissue staining, 20–30 μL of 3B4-Cy5 (30 μg/mL) in the incubation solution was applied onto the tissue slides and incubated for 1 h at room temperature. For a control, anti-mouse RAGE Mab (1/100, R&D Systems, MAB1179) was incubated overnight at 4°C, and tissues were treated with AlexaFluor-488-rabbit-anti-rat antibody (1/200, Abcam, ab169346) for 1 h at room temperature. Nuclei were counterstained with DAPI, and excess PBS was used for washing. Stained sections were mounted with cover slips using ProLong Gold antifade reagent (Invitrogen). Fluorescence microscopic images were taken with a Zeiss Observer Z1/Apotome 2 Microscope (Carl Zeiss) equipped with an EMCCD camera (Evolve 512 Delta, Photometrics, Tuscon, AZ), and the images were analyzed with ZEN 2011 software.

### Cellular immunofluorescence microscopy

The mouse pancreatic tumor cell (Panc02) was purchased from ATCC (American Type Culture Collection) and cultured in RPMI complete medium (ATCC) supplemented with 10% fetal bovine serum (FBS, Invitrogen) and penicillin (100 IU/mL) and streptomycin (100 μg/mL, Lonza) at 37 °C with 5% CO_2_. Cells were plated onto PLL-coated coverslips (BD Biocoat cellware) and incubated overnight. The medium was gently removed, and 3% paraformaldehyde (PFA) in PBS was added for fixation. After washing with PBS, cells were incubated with scFv-Cy5 (200 μL, 10 μg/mL) in the incubation buffer (3% BSA, 0.01% sodium azide, and 0.3% Tween in PBS) for 1h at room temperature. As a positive control, anti-mouse RAGE Mab (R&D Systems, MAB1179) was incubated with cells followed by Alexa Fluor 488 conjugated anti-mouse antibody (Abcam, ab169345). Sulfo Cy5-NHS solution was prepared with the equivalent concentration to the scFv-Cy5 and used as a control. Nuclei were counterstained with DAPI followed by washing with excess PBS. Coverslips were mounted on slides using ProLong Gold antifade reagent (Invitrogen) and kept at 4 °C. The same procedure was used for live cell labeling; after incubation with scFv-Cy5 (200 μL, 10 μg/mL) for 30 min at 37 °C, cells were fixed with 3% paraformaldehyde (PFA), and nuclei were counterstained with DAPI. Fluorescence microscopic images were taken with a Zeiss Observer Z1/Apotome 2 Microscope (Carl Zeiss) equipped with a Quantem 512SC camera, and the images were analyzed using ZEN 2011 software.

### Flow cytometry

Panc02 cells were harvested by trypsinization and fixed with 4% PFA. 5 x 10^4^ cells were used for each labeling condition (n ≥ 3). Cells were incubated with 3B4-Cy5 and M4-Cy5 (2 μg) in the incubation buffer (100 μL) for 30 min on ice. Anti-mouse RAGE Mab (1/100, R&D Systems, MAB1179) and Alexa Fluor 488 conjugated anti-mouse antibody (1/200, Abcam, ab169345) were sequentially incubated with cells for 30 min on ice. After each incubation, cells were washed with cold PBS three times. The same labeling conditions were used for live cell staining. Flow cytometric analysis was performed on a FACS LSR Fortessa flow cytometer (BD) using FACSDiva software provided by the University of Pittsburgh Cancer Institute Flow and Imaging Cytometry core facility, and the data was analyzed using VenturiOne software (Applied Cytometry).

### ^64^Cu labeling of scFv-NOTAs and biodistribution study

To NOTA-conjugated scFvs (200 μg, 3B4-NOTA and M4-NOTA), 74 MBq of ^64^Cu(OAc)_2_ in 200 μL of 0.1 M ammonium acetate (pH 6.5) was added and incubated in a thermomixer at 40°C, 550 rpm for 1 h. EDTA solution was added to stop the reaction (final concentration of 5 mM). ^64^Cu-labeled scFvs were purified by SEC (P-10, GE Healthcare) using 0.1 M ammonium acetate (pH 6.5) as a running buffer. Each fraction was tested with radio-TLC scanner, and pure fractions were collected and used for *in vivo* experiments.

A syngeneic model of pancreatic cancer, (Panc02) was established in female Balb/c nude mice (4–6 weeks of age, 16–22 g) from Taconic Lab Animals and Services. RAGE expressing mouse pancreatic tumor cells, Panc02 (1–2 x 10^6^), in 0.1 mL of PBS were injected subcutaneously onto the right flank of the mice. Tumors were allowed to grow for 3–4 weeks. Animals were monitored daily to minimize suffering and distress. The maximum tumor size was no greater than 8 × 8 mm. Any mouse that showed greater than 20% weight loss, lethargy, loss of appetite or diarrhea would have been euthanized; however, none of these characteristics were observed. There was no mortality or euthanasia outside the experimental plans. The specific activity of ^64^Cu-3B4-NOTA and ^64^Cu-M4-NOTA were 74–111 MBq/mg and 37–74 MBq/mg, respectively, and 185 to 300 pmol of scFvs (5–8 μg) were administered to mice intravenously divided randomly in groups. Mice were sacrificed 4 h post tracer injections, and organs were dissected for counting radioactivity using a γ-counter.

### Data analysis

All numeric data are presented as mean ± SEM. Statistical analysis was carried out using unpaired student’s *t*-test.

## Results

### Production, functionalization, and characterization of anti-RAGE scFv (3B4)

The two antibody fragments, termed 3B4 and M4, were expressed in BL21 *E*. *coli* and purified using immobilized metal affinity chromatography (IMAC, nickel-nitrilotriacetic acid). Analysis of the purified proteins on SDS-PAGE showed that both scFvs appeared as a single protein band at the molecular weight ~29 kDa (S1 Fig). Size exclusion chromatography verified that two scFvs were eluted at the corresponding retention time compared to the standard proteins. The purity of scFv from SEC was over 95% (S1 Fig).

For immunofluorescence detection of RAGE expression *in vitro* and *ex vivo*, sulfo-Cy5 NHS ester was conjugated with 3B4 and M4 via amide coupling reactions, which yielded 1.3 moles and 1.1 moles of sulfo-Cy5 per mole of 3B4 and M4, respectively. For the *in vivo* study, metal chelator conjugated scFvs were prepared by a bioconjugation from isothiocyanate-amine coupling reactions. The MALDI-TOF mass analysis confirmed that ~1–5 NOTAs were covalently conjugated to 3B4 and M4.

The functionalized scFvs were characterized using SDS-PAGE and size exclusion (SEC) chromatography, demonstrating the purity and stability of the optical dye and NOTA conjugates (S1 Fig). Moreover, the conjugated and parent scFvs were eluted at the same retention time in SEC chromatography indicating that the structural confirmation was preserved in chemical conjugation reactions. Fluorescence trace detection in SEC (λ_ex_/λ_em_ = λ_645 nm_ /λ_660 nm_) also confirmed the optically pure 3B4-Cy5.

**Fig 1:**
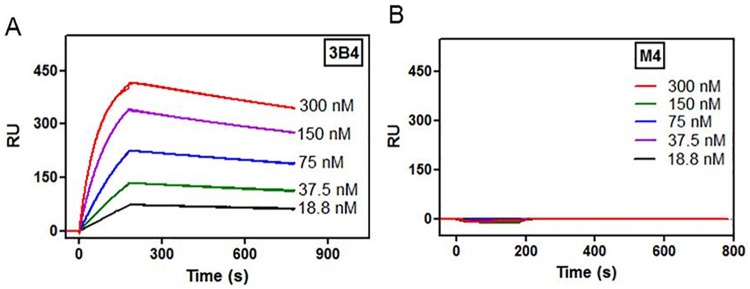
3B4 has a high affinity to mouse RAGE while the control M4 does not bind mouse RAGE. SPR sensorgrams of A. anti-RAGE scFv (3B4) and B. control scFv (M4) using the Langmuir 1:1 binding model.

The binding kinetics and affinity of RAGE specific antibody fragment was tested on the ectodomain of mouse RAGE (mRAGE)-immobilized gold chip in SPR studies. Evaluation with serial dilutions of 3B4 demonstrated a relatively strong affinity (K_D_ = 5.76 nM) binding affinity while no binding was observed were detected from the control scFv, M4 at the tested concentrations (Fig 1). Therefore, M4 was used as a non-binding control to mRAGE for further in vitro and in vivo studies.

### *In vitro* RAGE cell labeling

**Fig 2:**
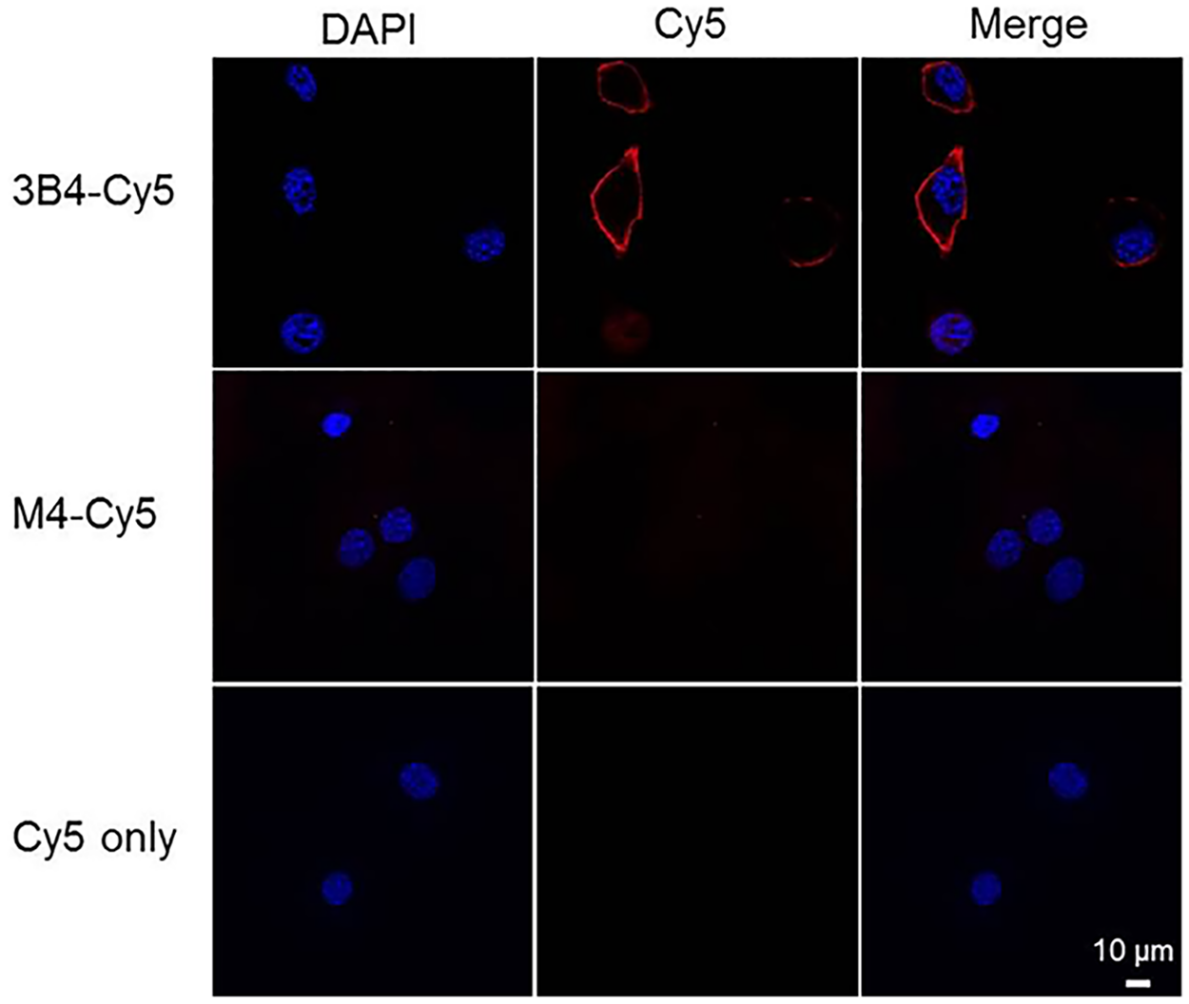
Confocal microscopic images of RAGE expressing Panc02 cells. Live cells were incubated with 3B4-Cy5 and M4-Cy5. After fixation, the nucleus was counterstained with DAPI (Scale bar = 10 μm).

RAGE expressing mouse pancreatic cancer cells (Panc02) were incubated with 3B4-Cy5. Fixed and live cell staining showed membrane RAGE localization of 3B4-Cy5 (Fig 2). Weak fluorescent signals were detected from the control experiments, both M4-Cy5 and Cy-5 only treatments. When live Panc02 cells were incubated with anti-mRAGE mAb, cellular internalization was observed, indicating membrane RAGE-mediated binding and subsequent cellular uptake (S2 Fig). However, in fixed cell labeling with mAb, Alexa Fluor 488 signals presented mostly on the cellular membrane. This *in vitro* immunofluorescence study demonstrates the high membrane RAGE specificity of 3B4-Cy5.

**Fig 3:**
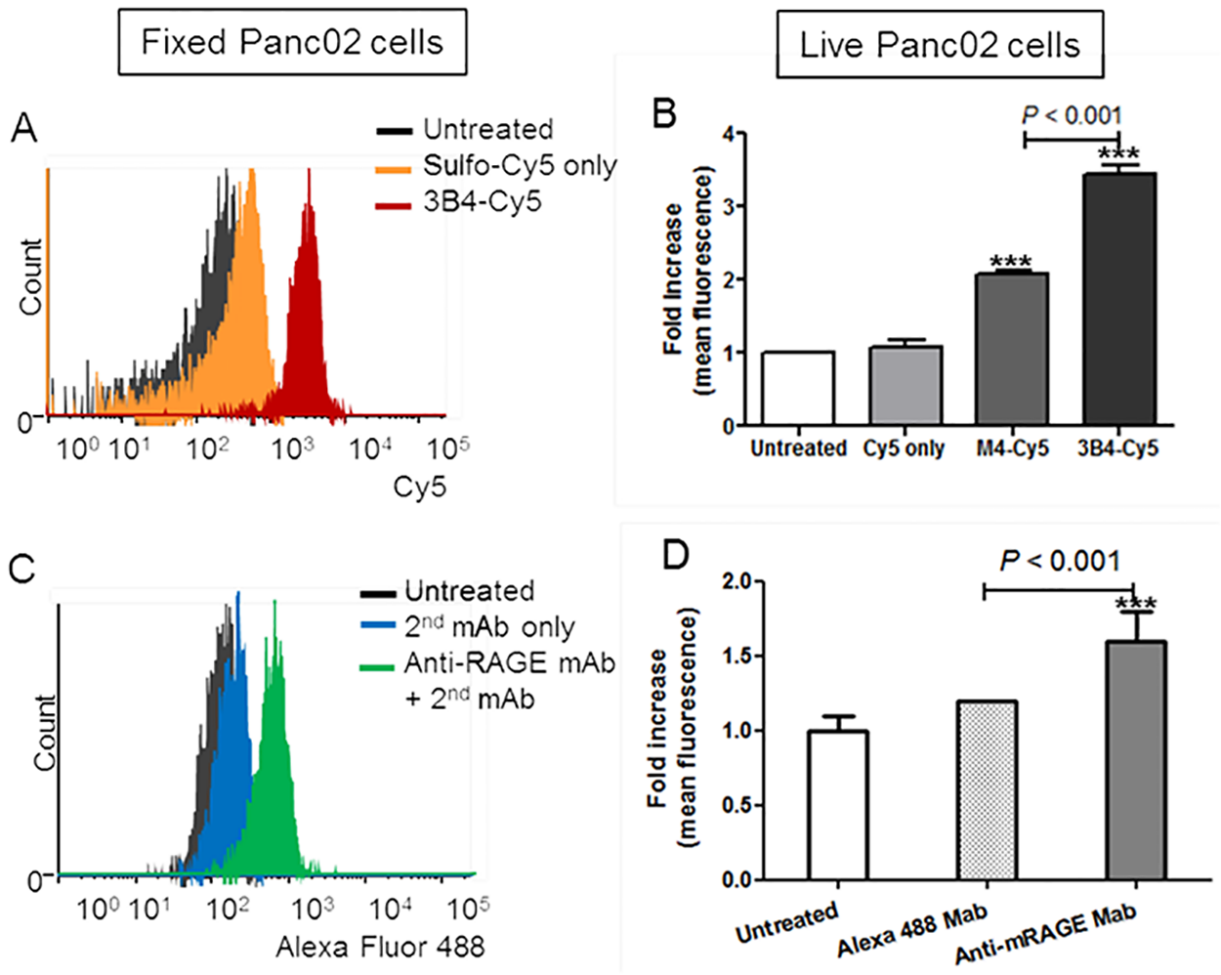
Flow cytometric analysis with fixed and live Panc02 cells demonstrate antibody-binding. (A) and (B) cells were incubated with anti-RAGE Mab followed by Alexa Fluor 488 secondary antibody. (C) and (D) cells were stained with 3B4-Cy5 and M4-Cy5 (n ≥ 3, ± SEM, *** *p* < 0.005 to the untreated cells).

Flow cytometry confirmed the epitope specificity of anti-RAGE scFv (3B4) in both fixed and live cell staining (Fig 3A and 3B). 3B4-Cy5 treated live cells showed 1.7-fold higher fluorescence than the M4-Cy5 treated cells (*P* < 0.001). Sulfo-Cy5 incubated cells produced low signals from nonspecific binding, which was at a similar level to the untreated cells in both fixed and live cell incubations. As a control, Panc02 cells were incubated with anti-RAGE mAb followed by Alexa Fluor 488-conjugated secondary antibody (Fig 3C and 3D). This quantitative flow cytometric analysis verified that 3B4-Cy5 binding to membrane RAGE is comparable to the monoclonal antibody treatment.

### *Ex vivo* tissue immunofluorescence analysis

**Fig 4:**
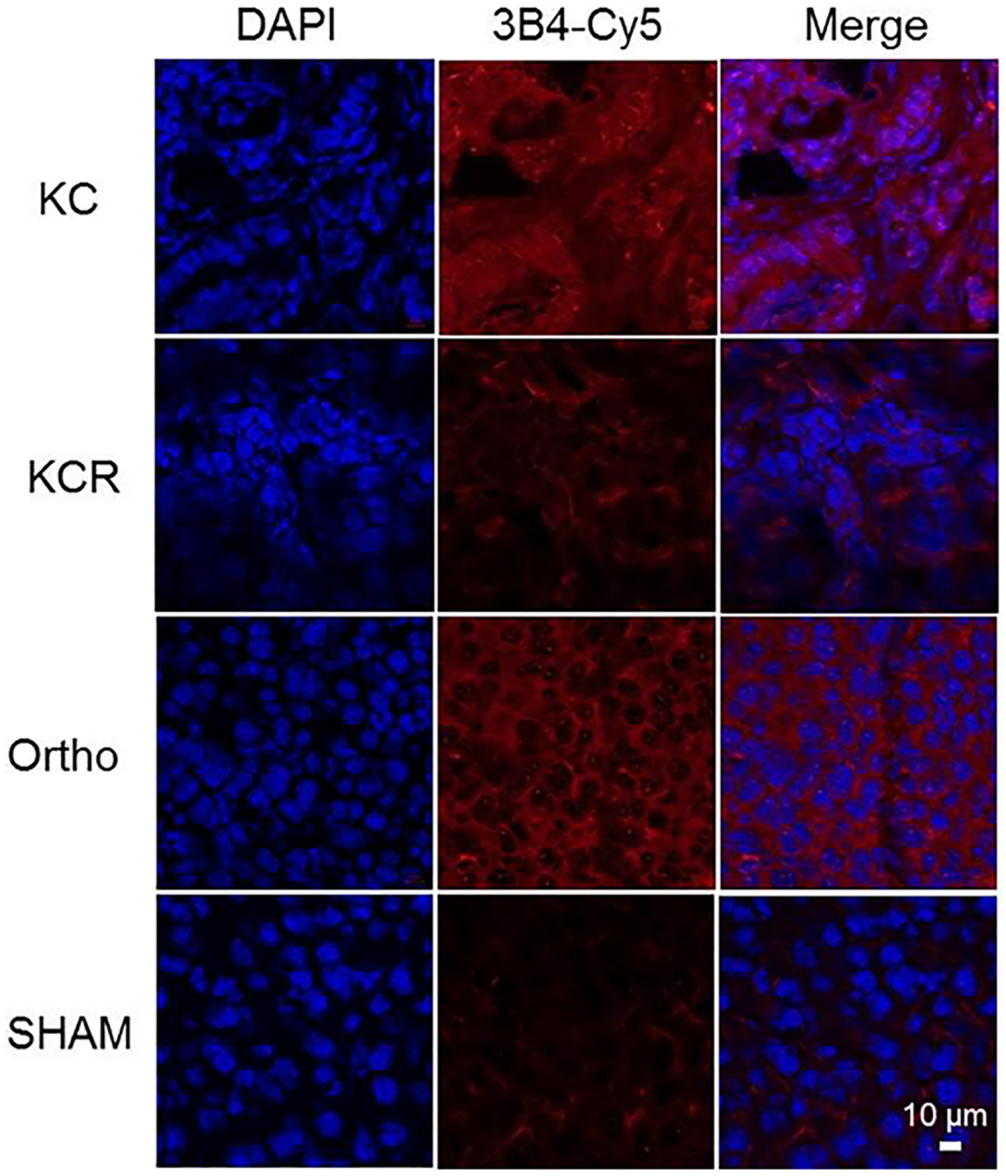
Tissue immunofluorescence staining of RAGE expression with 3B4-Cy5. The mouse pancreatic cancer specimens from KC, KCR, orthotopic, and SHAM mouse models were incubated with 3B4-Cy5. The nucleus was counterstained with DAPI (Scale bar = 10 μm).

To verify that 3B4 would recognize RAGE naturally expressed in tissues, pancreatic tumor tissues were obtained from the genetically engineered mouse models, KC and KCR. KC mice have a *Kras*^*G12D*^ expressed mutation which recapitulates the putative precursor lesions, human pancreatic intraepithelial neoplasias (PanINs), to invasive pancreatic cancer while KCR mice are KC mice with the RAGE gene universally ablated [5]. Additionally, pancreas tissues from orthotopically injected and sham control mice were also tested. Confocal microscopic images from 3B4-Cy5 staining showed RAGE expression-dependent fluorescence (Fig 4). KC mice and orthotopic pancreas tissue generated strong signals from 3B4-Cy5 binding to RAGE while the RAGE knockout KCR and the control sham mice showed relatively low fluorescent intensity. Sulfo-Cy5 only was incubated with tissues as a control, and weak fluorescence was detected from nonspecific tissue binding (S3 Fig). This confocal fluorescence imaging result was comparable to the double tissue staining with anti-RAGE mAb and AlexaFluor 488-rabbit-anti-rat antibody (S4 Fig), verifying 3B4 was functionally active and specific for RAGE.

### Biodistribution study

To validate RAGE targeting of anti-RAGE scFv, a biodistribution study was performed in a syngeneic pancreatic cancer model (Table 1). RAGE specific scFv (3B4) and its non-binding control scFv (M4) were conjugated with *p*SCN-Bn-NOTA via a thiourea linker. ^64^Cu labeling followed by SEC purification (P-10, GE Healthcare) yielded >98% pure fractions. The specific activity of ^64^Cu-3B4-NOTA and ^64^Cu-M4-NOTA were 74–111 MBq/mg and 37–74 MBq/mg, respectively, and 185 to 300 pmol of scFvs (5–8 μg) were administered to mice intravenously. The results are expressed as per cent injected dose per gram tissue (%ID/g) and the mice were healthy with no mortality during the growth of the tumors. No adverse events were obvserved. ^64^Cu-3B4-NOTA showed higher tumor accumulation compared with the ^64^Cu-M4-NOTA treated group, but statistical difference was not achieved. However, statistically significant tumor/blood ratios of ^64^Cu-3B4-NOTA relative to the non-binding control were achieved (3B4: 1.65 ± 0.45, M4: 0.64 ± 0.15, p < 0.05, unpaired t-test). Moreover, statistically higher accumulation of ^64^Cu-3B4-NOTA relative to the non-binding control was observed in RAGE-enriched kidney, liver, lung, spleen, and bone (presumably marrow) [14–17].

**Table 1:** Biodistribution analysis of ^64^Cu-3B4-NOTA and ^64^Cu-M4-NOTA at 4 h p.i. in Balb c/nude mice bearing Panc02 tumors.

	^64^Cu-3B4-NOTA	^64^Cu-M4-NOTA
Blood	0.65 ± 0.05	1.47 ± 0.13***
Kidney	83.0 ± 5.66	45.0 ± 2.92***
Liver	43.14 ± 2.45	12.27 ± 1.44***
Lung	3.07 ± 1.07	1.34 ± 0.19^#^
Spleen	12.18 ± 0.83	4.14 ± 0.43***
Muscle	0.27 ± 0.05	0.15 ± 0.02**
Heart	0.72 ± 0.06	0.75 ± 0.10
Bone	3.39 ± 0.21	1.73 ± 0.21***
Tumor	1.06 ± 0.14	0.95 ± 0.14
Stomach	0.50 ± 0.08	0.28 ± 0.03**
Pancreas	0.43 ± 0.03	0.53 ± 0.05^#^

n = 5/group, mean ± SEM,

** *p* < 0.05,

*** *p* < 0.005,

^#^ *p* < 0.1

## Discussion

RAGE, post-embryologically, is expressed at only very low levels in most cell types other than the lung [12]. Under pathological states, RAGE is upregulated in a number of pathogenic diseases such as neurodegeneration, diabetes, vascular disease, and cancer [18]. RAGE can also be expressed on a variety of cell types such as macrophages, T and B lymphocytes, endothelial cells, dendritic cells, and fibroblasts as well as cancer cells at the end stage of tumor development [7]. Importantly, the overexpression of RAGE is a direct link to the survival of premalignant epithelial precursors and tumor cells in PDAC [5]. RAGE ligands, AGE, HMGB1, and S100 protein family, secreted from cancer cells and leukocytes interact with RAGE and other receptors and regulate further tumor progression [7]. Oligomeric RAGE ligand engagement upregulates membrane RAGE expression and cluster formation on the cell surface. Serum levels of AGE and HMGB1 are elevated in pancreatic cancer patients. AGE/RAGE binding and subsequent internalization is essential to trigger proinflammatory responses via cellular activation [19, 20]. Thus, the accumulated evidence indicates that the RAGE ligand is a promising biomarker to assess the progression of PDAC. Therefore, a radiolabeled high affinity ligand for RAGE could serve as a molecular imaging agent that could aid in the non-invasive diagnosis of PDAC [21–27].

Owing to their high target specificity and affinity, monoclonal antibodies have been used for *in vivo* detection of tumor associated antigens or cell surface markers for non-invasive diagnosis of cancer. The intact antibody (150 kDa) remains in the circulation for days to weeks (t_1/2_ 1–3 wk), which hampers effective temporal PET imaging. The smallest functional binding unit of antibody, a single chain Fv (25–30 kDa) is advantageous in terms of rapid blood clearance and deep tumor penetration [28]. Moreover, an scFv lacking the Fc domain does not induce Fcγ receptor-mediated effector functions. Here, a single chain Fv, 3B4, was investigated for detection of RAGE expressing pancreatic tissues *in vitro* and *in vivo*. 3B4 is an scFv with an affinity matured CDR3 of the anti-RAGE-Mab (XT-M4) derived from the rat [29]. It was raised against the N-terminal ectodomain of rat RAGE and binds the V-domain [30, 31].

To demonstrate that 3B4 was cross reactive with mouse-RAGE, it was conjugated to a fluorescent dye for immunofluorescence microscopy and flow cytometry of RAGE-enriched mouse pancreatic tissues. 3B4 strongly stained mouse pancreatic tissues that upregulate RAGE as well as orthotopic tumor implants and cell lines, indicating that 3B4 is functionally active and can tolerate the modification of Lys residues (Figs 2, 3, and 4). In addition, the immunofluorescence cell staining suggests that anti-RAGE scFv (3B4) is a non-internalizing antibody fragment (Fig 2). The RAGE ligand binding and subsequent cellular internalization is a key molecular event in innate/adaptive immune responses, cancers, diabetes, and neurodegenerative disease [8, 9, 32, 33]. In particular, the treatment with rat anti-RAGE mAb increased the survival in mouse models of sepsis and systemic infection by inhibiting RAGE interactions with its ligands [34]. Thus, anti-RAGE scFv (3B4) could be used as an immune-suppressor that impedes immune cell adhesion and infiltration. This RAGE specific antibody fragment (3B4) shows therapeutic potential in other RAGE-associated diseases and cancer and retains those properties of the parent antibody [31].

The binding kinetics of antibody is a critical factor that governs target tumor retention for imaging as well as therapy. However, there is not always a direct correlation between high affinity *in vitro* and potency *in vivo*, which is determined by the pharmacokinetic profile and receptor-mediated agonism/antagonism [35, 36]. Tumor retention did not increase quantitatively with the enhancements in affinity beyond 10^−9^ M *in vivo* biodistribution study, showing that extremely high binding affinity impedes tumor localization and retention [37]. When the V*H* and V*L* domains of 3B4 were incorporated into the IgG format, the association and dissociation rate constants of 1.10 ×10^6^ M^-1^s^-1^ and 2.90 ×10^−4^ s^-1^ respectively were measured, resulting in a relatively high affinity (K_*D*_ = 0.29 ×10^−9^ M) [13]. The association rate constant (*k*_a_ = 6.2 ×10^4^ M^-1^s^-1^) of monovalent anti-RAGE scFv (K_*D*_ = 5.8 ×10^−9^ M) decreased to 17.6-fold with 1.2-fold increase in off-rate (*k*_d_ = 3.6 ×10^−4^ s^-1^) compared to the parental monoclonal antibody. While this is a considerable decrease in affinity, it is still suitable for *in vivo* RAGE targeted imaging.

The tumor accumulation of ^64^Cu-3B4-NOTA in Panc02 tumors was relatively low and not statistically different from that of the non-binding control; we did observe, however, statistical differences between ^64^Cu-3B4-NOTA and ^64^Cu-M4-NOTA in tumor-to-muscle ratios. Moreover, ^64^Cu-3B4-NOTA uptake was significantly higher than the non-binding control in tissues known to be enriched in RAGE, kidney, liver, spleen and bone [12, 14–17]. These findings indicate that ^64^Cu-3B4-NOTA is functionally active and that the tissue accumulations are, in part, RAGE mediated.

One reason for the low tumor accumulations is because of the relatively rapid blood clearance through the kidney, which is well-known when utilizing the scFv antibody format. Additionally, kidney, liver, and spleen functioned as an antigen sink and depleted the tracer from circulation thus further reducing tumor accumulation [38, 39]. In future work, we will reformat the scFv into bivalent antibody fragments, either as a diabody (55 kDa, t_1/2_ 2–5 h) or a minibody (75 kDa, t_1/2_ 5–12 h) with pharmacokinetics that will improve target to non-target tissue ratios and will be amenable to serial imaging of RAGE expression in aggressive animal models such as syngeneic orthotopic PDAC models [40, 41]. We may incorporate pre-dosing in the tumor-bearing animal models with unlabeled RAGE-antibody to pre-saturate off-target RAGE receptors prior to imaging studies. This strategy can be highly effective, preventing rapid depletion of tracers from the serum before they can localize to the target tissue receptors [38].

High-mobility group box 1 (HMGB1) is a major ligand both inducing expression and binding to RAGE, playing a major role in cancer development [41]. The ability to target RAGE with PET reagents may also depend on receptor occupancy with HMGB1 and other ligands [42]. Ligand binding of RAGE promotes an ERK-dependent signaling series of events, resulting in production of reactive oxygen species (ROS), inflammation, cellular proliferation/apoptosis, and upregulation of RAGE expression. Although small-molecule inhibitors of RAGE have been developed for therapeutic intervention, they are not very far advanced in the clinic. Our colleagues have demonstrated that RAGE is a major mediator of pulmonary inflammatory responses [43]. RAGE is most highly expressed in lung tissue and expression there may limit the ability of PET imaging for lesions located in the lung. Carbenoxolone, an HMGB1 antagonist, limit metastatic seeding in the lungs [44], mediated by downregulation of the adhesion molecule Intercellular Adhesion Molecule 1 (ICAM1). Our recent work demonstrates that RAGE ablation in murine models of accelerated pancreatic carcinogenesis with specific ablation of HMGB1 expression in the emergent malignant cells can be obviated by knocking out global RAGE expression but not TLR9 [45]. This suggests that paracrine production of HMGB1 by inflammatory cells mediates its major effect through RAGE expression, again supporting the important role of the HMGB1/RAGE axis in cancer.

In summary, we have developed and evaluated a RAGE-specific scFv, 3B4, for evaluation as an imaging agent for RAGE expression in PDAC. The scFv was affinity matured from a parent Mab that had shown *in vivo* efficacy as a RAGE antagonist. This scFv, when labelled with a fluorescent dye, readily stained murine and human RAGE positive tissues in immunofluorescence microscopy as well as cells in a receptor specific manner. 3B4 had a high affinity for the RAGE receptor that is necessary for a molecular imaging agent and modifications of the ɛ-amino groups for Lys or the N-terminal amine were not detrimental to affinity. The biodistribution of the radiolabeled B4 indicated uptake in RAGE-enriched tissues, which, in conjunction with the relatively rapid blood clearance, confounded analysis of RAGE-mediated tumor uptake in the syngeneic animal model. However, these results warrant future investigations of the scFv 3B4 applied in larger molecular weight antibody fragments such as minibodies or diabodies for further investigations to formulate a non-invasive diagnostic imaging agent for patients with PDAC.

## Supporting information

S1 Checklist ARRIVE checklist. (PDF)

S1 Fig Characterization of scFvs using SDS-PAGE (A) and SEC (B and C). (PDF)

S2 Fig Confocal microscopic images of RAGE expressing Panc02 cells. Fixed and live cells were incubated with anti-RAGE Mab followed by Alexa Fluor 488 secondary antibody. The nucleus was counterstained with DAPI (Scale bar = 10 μm).
(PDF)

S3 Fig Tissue immunofluorescence staining using sulfo-Cy5. Mouse pancreatic cancer specimens from KC, KCR, orthotopic, and SHAM mouse models were incubated with sulfo-Cy5. The nucleus was counterstained with DAPI (Scale bar = 10 μm).
(TIF)

S4 Fig Tissue immunofluorescence staining of RAGE expression using anti-RAGE Mab. Mouse pancreatic cancer specimens from KC, KCR, orthotopic, and SHAM mouse models were incubated with anti-RAGE Mab followed by Alexa Fluor 488 secondary antibody consecutively. The nucleus was counterstained with DAPI (Scale bar = 10 μm).
(TIF)
