# Supplementary figures and images for: RAGE-specific single chain Fv for PET imaging of pancreatic cancer
Source: PLoS One. 2018 Mar 12;13(3):e0192821. doi: 10.1371/journal.pone.0192821 (PMC5846720; doi:10.1371/journal.pone.0192821)

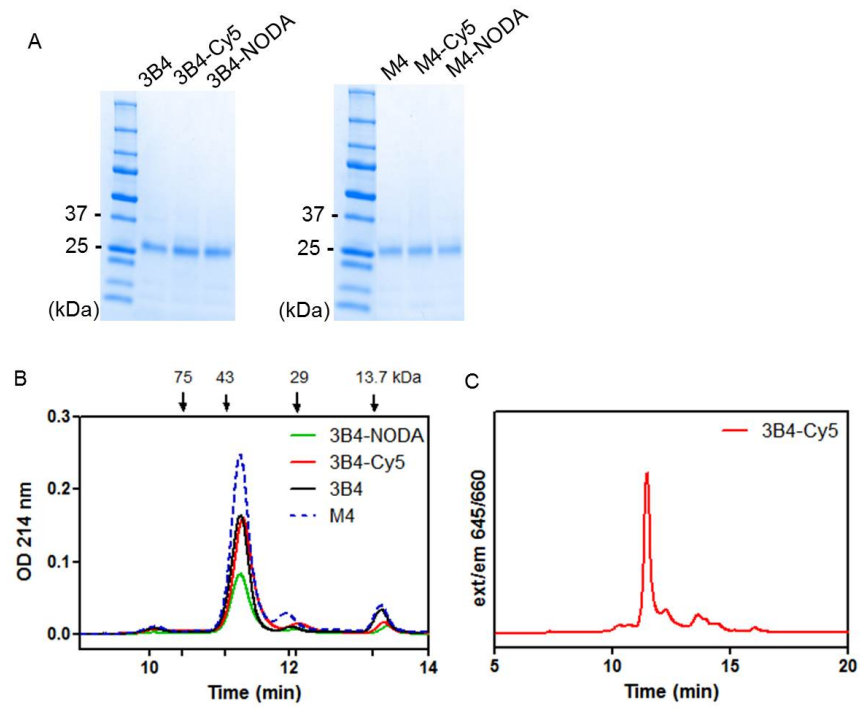

**S1Fig. Characterization of scFvs using SDS-PAGE (A) and SEC (B and C).**

Supplement: S1 Fig — (PDF) [file pone.0192821.s002.pdf]

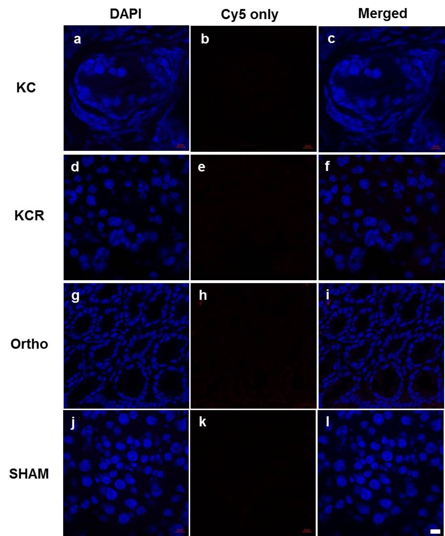

Supplement: S3 Fig — Mouse pancreatic cancer specimens from KC, KCR, orthotopic, and SHAM mouse models were incubated with sulfo-Cy5. The nucleus was counterstained with DAPI (Scale bar = 10 μm). (TIF) [file pone.0192821.s004.tif]

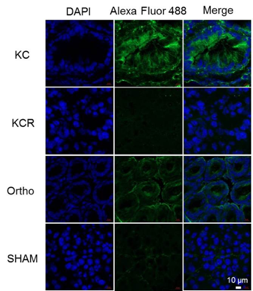

Supplement: S4 Fig — Mouse pancreatic cancer specimens from KC, KCR, orthotopic, and SHAM mouse models were incubated with anti-RAGE Mab followed by Alexa Fluor 488 secondary antibody consecutively. The nucleus was counterstained with DAPI (Scale bar = 10 μm). (TIF) [file pone.0192821.s005.tif]
