# Supplementary material for: RAGE-specific single chain Fv for PET imaging of pancreatic cancer
Source: PLoS One. 2018 Mar 12;13(3):e0192821. doi: 10.1371/journal.pone.0192821 (PMC5846720; doi:10.1371/journal.pone.0192821)
Supplement: S2 Fig — Fixed and live cells were incubated with anti-RAGE Mab followed by Alexa Fluor 488 secondary antibody. The nucleus was counterstained with DAPI (Scale bar = 10 μm). (PDF) [file pone.0192821.s003.pdf]

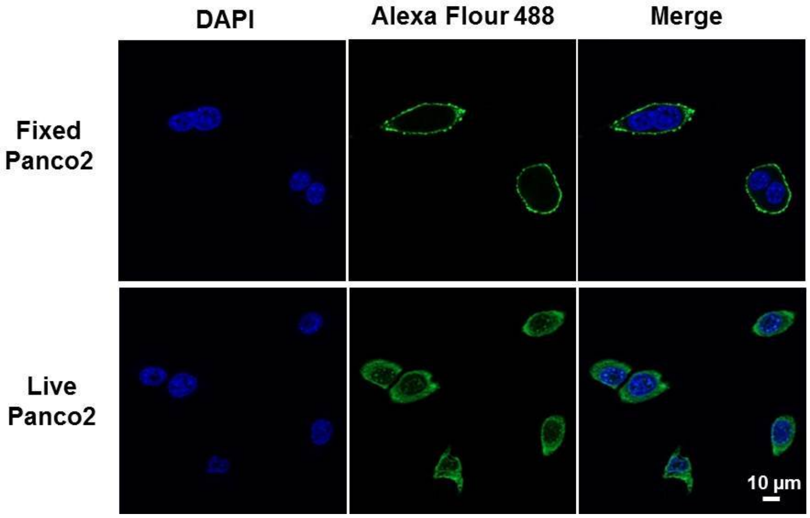

S2 Fig. Confocal microscopic images of RAGE expressing Panc02 cells. Fixed and live cells were incubated with anti-RAGE Mab followed by Alexa Fluor 488 secondary antibody. The nucleus was counterstained with DAPI (Scale bar = 10 µm).
